# Clinical Features and Outcomes of COVID-19 Patients with Acute Kidney Injury and Acute Kidney Injury on Chronic Kidney Disease

**DOI:** 10.14336/AD.2021.1125

**Published:** 2022-06-01

**Authors:** Zhifeng Xu, Yuanyuan Zhang, Chun Zhang, Fei Xiong, Jianduan Zhang, Jing Xiong

**Affiliations:** ^1^Department of Nephrology, Union Hospital, Tongji Medical College, Huazhong University of Science and Technology, Wuhan, China.; ^2^Department of Maternal and Child Health, School of Public Health, Tongji Medical College, Huazhong University of Science and Technology, Wuhan, China.; ^3^Department of Nephrology, Wuhan No. 1 Hospital, Wuhan, China

**Keywords:** COVID-19, SARS-CoV-2, AKI, AKI on CKD, retrospective study

## Abstract

COVID-19 emerged in Wuhan in December 2019 and soon became a worldwide pandemic. We collected and analyzed the data from 1077 patients with COVID-19 who were admitted to the west campus of Wuhan Union Hospital from January 16 to April 16, 2020. Sixty (5.6%) of the 1077 COVID-19 patients were diagnosed with acute kidney injury (AKI) during hospitalization, and 18 of them (30%) had AKI on chronic kidney disease (AKI/CKD). COVID-19 patients with AKI had a worse prognosis, with higher intensive care unit (ICU) admission (28.3%) and fatality (65%) rates than patients without AKI (3.4% and 10.7%, respectively). Among the COVID-19 patients, AKI was more likely to occur in male patients, the elderly, patients with more severe disease states and those with comorbidities (such as hypertension, diabetes, coronary heart disease (CHD), chronic obstructive pulmonary disease (COPD) and CKD). COVID-19 patients with AKI were more likely to develop respiratory failure, gastrointestinal bleeding, acute liver injury, acute myocardial injury, heart failure, acute respiratory distress syndrome (ARDS), cerebrovascular accident, and disseminated intravascular coagulation (DIC) than those without AKI. Compared with patients without AKI, COVID-19 patients with AKI had lower platelet counts, lymphocyte counts, albumin levels and serum calcium levels but had elevated leukocyte counts, neutrophil counts and serum potassium levels. Inflammatory indicators, such as C-reactive protein (CRP), interleukin-6 (IL-6), and procalcitonin (PCT), were significantly higher in patients with AKI than in those without AKI. COVID-19 patients with AKI also exhibited a longer prothrombin time (PT), a longer activated partial thromboplastin time (APTT), and a higher D-dimer level than those without AKI. Survival analysis revealed that COVID-19 patients with AKI had a reduced survival rate compared with those without AKI. Furthermore, COVID-19 patients with AKI/CKD had a lower survival rate than those with AKI or CKD only. Multiple logistic regression indicated that the predictors of AKI in COVID-19 patients included complications, such as respiratory failure and acute myocardial injury, and higher creatinine and PCT levels during hospitalization.

Pneumonia cases of unknown origin emerged in Wuhan in December 2019, and a novel coronavirus was quickly identified as the cause [1, 2]. The novel coronavirus was named severe acute respiratory syndrome coronavirus 2 (SARS-CoV-2) and exhibited phylogenetic similarity to SARS-CoV. The World Health Organization (WHO) officially named the disease caused by the virus coronavirus disease 2019 (COVID-19) [3, 4]. COVID-19 is a highly contagious disease that created a pandemic, with 258,164,425 cases and 5,166,192 deaths reported worldwide as of November 24, 2021 [5].

SARS-CoV-2 induces inflammation in lung tissue and leads to acute respiratory distress syndrome (ARDS), which is similar to Middle East respiratory syndrome coronavirus (MERS-CoV) and SARS-CoV [6]. The lung is the main target organ of SARS-CoV-2, and pneumonia is the initial clinical sign in most COVID-19 patients. However, this virus causes multiple organ damage [7]. Organs such as the heart, liver, and kidney may be involved in COVID-19 [8-11]. Early clinical research reports from China, Italy and the United States have indicated that the rate of acute kidney injury (AKI) in COVID-19 patients ranges from 0.5% to 36.3% [1, 4, 12, 13]. Our previous studies found that the pathological manifestations of kidney injury in COVID-19 patients included diffuse proximal tubule injury with brush border loss, nonisometric vacuolar degeneration, frank necrosis, prominent erythrocyte aggregates obstructing the lumen of capillaries without platelet or fibrinoid material and clusters of coronavirus particles in the tubular epithelium and podocytes [14]. Other studies also observed severe acute tubular necrosis, the SARS-CoV-2 NP antigen, and vacuoles containing the virus accumulated in kidney tubules [15].

In the current study, we collected and analyzed data from patients with COVID-19 who were admitted to the west campus of Wuhan Union Hospital. We investigated the morbidity of AKI and AKI on chronic kidney disease (AKI/CKD) among COVID-19 patients to describe the clinical features and outcomes of these patients and identify the risk factors associated with AKI development.

## MATERIALS AND METHODS

### Study design and participants

This was a retrospective study performed on the west campus of Wuhan Union Hospital, which was one of the major general hospitals assigned by the government for treating COVID-19 patients during the outbreak in Wuhan. We enrolled 1077 patients with COVID-19 who were admitted to the hospital from January 16 to April 16, 2020. The diagnosis of COVID-19 followed WHO interim guidance [16]. The inclusion criteria were based on laboratory confirmation of SARS-CoV-2 infection. SARS-CoV-2 RNA was positive in samples from nasopharyngeal swabs using reverse transcription polymerase chain reaction (RT-PCR) and next-generation sequencing analysis. The exclusion criteria were as follows: (1) patients clinically diagnosed with COVID-19; (2) pediatric patients; (3) patients with a history of renal transplantation. The sample size was not formally determined, and all patients who met the inclusion criteria were included.

This study was approved by the Ethics Committee of Wuhan Union Hospital, Tongji Medical College, Huazhong University of Science and Technology, Wuhan, China before study inception. Written informed consent was waived given the urgency and unprecedented nature of the COVID-19 pandemic.

### Data collection

Patient information, including demographic characteristics, clinical symptoms, laboratory and chest computed tomography (CT) examinations, treatments, complications, and outcomes, was obtained from electronic medical records of the hospital information system and patient interviews. All the data were independently reviewed and validated by a group of physicians. Laboratory data included the results of the following: routine blood tests; lymphocyte subsets; liver, renal and coagulation function tests; lactate dehydrogenase (LDH), creatine kinase, D-dimer, high-sensitivity CRP, PCT, electrolytes, and IL-6; creatine kinase serum levels; and the erythrocyte sedimentation rate. The thresholds of these measures were provided by our laboratory. Chest CT scans were obtained from the initial diagnosis. Treatments for COVID-19, including the administration of antibiotics and antivirals, oxygen therapy and mechanical ventilation, complications, and outcomes during admission to the hospital, were also included.

### Definitions

The outcomes in our study included death and discharge. The criteria for discharge of COVID-19 patients were set according to the Diagnosis and Treatment Protocol for COVID-19 by the National Health Commission of China (trial version 7) [17]. Follow-up time began at the date of admission and ended at the date of outcome for each patient. Variables such as age, sex, COVID-19 severity, basic illness (hypertension, diabetes, CHD, COPD, CVD, or tumors), high-sensitivity CRP, the lymphocyte count, D-dimer, and LDH were associated with poor outcomes of COVID-19 patients. Those exposures were considered adjusted variables when we used Cox regression models to compare the survival curves [1, 4, 18].

The severity of COVID-19 pneumonia was staged according to the Diagnosis and Treatment Protocol for COVID-19 by the National Health Commission of China (Trial Version 7) [17]. AKI was identified according to Kidney Disease: Improving Global Outcomes [19] as an increase in serum creatinine ≥0.3 mg/dl (≥26.5 µmol/l) within 48 h and an increase in serum creatinine ≥1.5 times the baseline within the previous 7 days during hospitalization or before admission (Creatinine decreased ≥26.5 µmol/L within 48 h or decreased by ≥1.5 times admission creatinine within 7 days). AKI was further staged as AKI stage 1, 2, or 3 when the serum creatinine level increased 1.5- to 1.9-fold, 2.0- to 2.9-fold, or 3-fold or more, respectively, within 7 days. Renal recovery of AKI was defined as creatinine returning to the normal range when patients were discharged. Diagnostic thresholds for CKD were a glomerular filtration rate (GFR) less than 60 mL/min per 1.73 m^2^ and an albumin-creatinine ratio (ACR) of 30 mg/g or greater at least 3 months before admission [20]. We evaluated CKD-related complications, such as anemia and mineral and bone disorders, when GFR was only measured once and matched GFR<60 mL/min per 1.73 m^2^ in outpatients.

**Table 1:** General Characteristics of 1077 patient between AKI and Non-AKI group.

Variables	Non-AKI (n=1017)	AKI (n=60)	Total (n=1077)	*P-*value	Missing
Age	57.5±14.1	67.0±14.3	58.0±14.3	<0.001	0
Sex					
Female	501/1017 (49.3%)	14/60 (23.3%)	515/1077 (47.8%)	<0.001	0
Male	516/1017 (50.7%)	46/60 (76.7%)	562/1077 (52.2%)		
Hospitalization time (day)	22.6±14.0	18.3±13.8	22.3±14.1	0.009	11
Systolic BP (mm Hg)	131.2±17.9	133.1±22.1	131.3±18.2	0.706	69
Diastolic BP (mm Hg)	81.7±12.8	79.0±12.8	81.5±12.8	0.179	72
COVID-19 Severity					
Mild	23/1017 (2.3%)	0/60 (0%)	23/1077 (2.1%)	<0.001	0
Moderate	553/1017 (54.4%)	10/60 (16.7%)	563/1077 (52.3%)		
Severe	342/1017 (33.6%)	10/60 (16.7%)	352/1077 (32.7%)		
Critically severe	99/1017 (9.7%)	40/60 (66.7%)	139/1077 (12.9%)		
Basic illness					
Hypertension	296/1017 (29.1%)	21/60 (35.0%)	317/1077 (29.4%)	0.330	0
Diabetes	182/1017 (17.9%)	18/60 (30.0%)	200/1077 (18.6%)	0.019	0
CHD	97/1017 (9.5%)	8/60 (13.3%)	105/1077 (9.7%)	0.335	0
COPD	18/1017 (1.8%)	5/60 (8.3%)	23/1077 (2.1%)	0.003	0
CVD	18/1017 (1.8%)	4/60 (6.7%)	22/1077 (2.0%)	0.033	0
Tumor	37/1017 (3.6%)	4/60 (6.7%)	41/1077 (3.8%)	0.399	0
ICU admission	35/1017 (3.4%)	17/60 (28.3%)	52/1077 (4.8%)	<0.001	0
Death	109/1017 (10.7%)	39/60 (65%)	148/1077 (13.7%)	<0.001	0
Complications					
Respiratory failure	101/1017 (9.9%)	42/60 (70%)	143/1077 (13.3%)	<0.001	0
Gastrointestinal bleeding	14/1017 (1.4%)	8/60 (13.3%)	22/1077 (2%)	<0.001	0
Acute liver dysfunction	318/1016 (31.3%)	38/60 (63.3%)	356/1076 (33.1%)	<0.001	1
Acute myocardial injury	49/1014 (4.8%)	32/59 (54.2%)	81/1073 (7.5%)	<0.001	4
Heart failure	34/1017 (3.3%)	23/60 (38.3%)	57/1077 (5.3%)	<0.001	0
ARDS	54/1016 (5.3%)	30/60 (50%)	84/1076 (7.8%)	<0.001	1
Cerebrovascular accident	9/1016 (0.9%)	4/60 (6.7%)	13/1076 (1.2%)	0.004	1
Bacteremia	15/1017 (1.5%)	6/60 (10%)	21/1077 (1.9%)	<0.001	0
DIC	9/1016 (0.9%)	11/60 (18.3%)	20/1076 (1.9%)	<0.001	1
CKD	83/1017 (8.2%)	18/60 (30.0%)	101/1077 (9.4%)	<0.001	0
hematuria	77/235 (32.8%)	18/27 (66.7%)	95/262 (36.3%)	0.001	815
proteinuria	27/232 (11.6%)	14/26 (53.8%)	41/258 (15.9%)	<0.001	819
ACEI/ARB history	45/742 (6.1%)	2/46 (4.3%)	47/788 (6.0%)	0.876	289

BP, blood pressure; CKD, Chronic kidney disease; CHD, coronary heart disease; COPD, Chronic obstructive pulmonary disease; AKI, acute kidney injury; CVD, cerebrovascular disease; ARDS, Acute Respiratory Distress Syndrome; DIC, disseminated intravascular coagulation; LDH, Lactate dehydrogenase; UA, uric acid; PCT, Procalcitonin; SO2, Blood oxygen saturation; PLT, Blood platelets; CRP, C-reactive protein.

### Statistical analysis

Categorical variables were summarized as percentages, and continuous variables were expressed as the means and standard deviation (SD). Between-group differences for continuous variables were examined using two-sample t-test, analysis of variance (ANOVA), or nonparametric methods such as the Wilcoxon rank sum test and Kruskal-Wallis test. For categorical variables, distributional differences were tested using chi-squared test or Fisher’s exact probability test as appropriate. Multiple logistic regression using the backward automatic variable selection method was performed based on 10 multiple imputed datasets to examine factors associated with the onset of AKI. A significance level of 0.1 was used to screen variables for inclusion in the multiple regression analysis [21, 22]. Variables, such as admission creatinine, treatments in the hospital, and ICU admission, were excluded for collinearity or professional reasons (some treatments and situations, such as ICU admission, often occurred after AKI occurrence).

**Table 2:** Laboratory examination of 1077 patients between AKI and non-AKI group.

Variables	Non-AKI (n=1017)	AKI (n=60)	Total (n=1077)	*P*-value	Missing
Hemoglobin (g/L)	126.4±19.3	128.2±20.9	126.5±19.4	0.777	8
PLT (10^9^/L)	229.0±85.5	204.5±91.3	227.6±85.9	0.012	7
WBC count (10^9^/L)	6.5±3.2	8.4±4.5	6.6±3.4	<0.001	9
Neutrophil count (10^9^/L)	5.0±13.7	7.0±4.4	5.1±13.4	<0.001	11
Lymphocyte count (10^9^/L)	1.8±8.0	0.9±0.6	1.8±7.8	<0.001	8
Eosinophil count(10^9^/L)	0.2±1.2	0.0±0.1	0.1±1.1	<0.001	7
Albumin (g/L)	33.9±6.3	29.6±5.1	33.6±6.3	<0.001	6
Globulin (g/L)	31.0±14.1	34.1±7.3	31.2±13.8	<0.001	13
ALT (U/L)	43.8±41.9	54.9±46.2	44.4±42.2	0.032	5
AST (U/L)	33.6±25.5	63.7±76.7	35.3±31.4	<0.001	5
LDH (U/L)	254.4±147.6	444.1±191.0	265.1±156.5	<0.001	11
CK (U/L)	107.9±189.0	304.7±408.6	119.3±212.7	<0.001	126
CK-MB (U/L)	12.5±9.4	19.0±13.7	12.9±9.8	<0.001	111
HsTnI (ng/L)	53.9±512.1	251.9±740.4	69.4±535.5	<0.001	479
BNP (pg/mL)	72.3±213.1	168.6±252.8	81.4±218.7	<0.001	593
Admission creatinine (μmol/L)	71.2±60.4	126±123.4	74.2±66.7	<0.001	6
Peak creatinine (μmol/L)	74.3±61.0	219.6±150.9	82.6±77.0	<0.001	26
Serum urea nitrogen (mmol/L)	5.3±3.2	12.3±11.8	5.7±4.4	<0.001	7
UA(μmol/L)	277.0±108.7	351.6±227.7	281.2±119.7	0.029	12
Blood natrium (mmol/L)	141.5±57.3	140.2±8.1	141.4±55.7	0.722	13
Blood Potassium (mmol/L)	4.1±4.6	4.2±0.7	4.1±4.4	0.029	12
Blood calcium (mmol/L)	2.1±4.2	1.9±0.2	2.1±4.0	<0.001	10
Fasting blood glucose (mmol/L)	7.4±22.6	9.5±5.7	7.5±22.0	<0.001	19
PT (s)	13.9±7.8	15.2±7.2	14.0±7.8	0.001	65
APTT (s)	38.1±20.5	39.7±7.8	38.2±20.0	0.005	66
D-dimer (ug/mL)	1.4±2.2	3.7±3.2	1.6±2.4	<0.001	149
High-sensitivity CRP (mg/L)	26.3±38.2	71.3±51.5	29.0±40.5	<0.001	124
PCT (ng/mL)	0.2±0.9	3.2±14.1	0.4±3.7	<0.001	240
IL6 (pg/mL)	21.8±252.1	385.4±1279.1	35.1±349.0	<0.001	666

LDH, Lactate dehydrogenase; UA, Uric acid; PCT: Procalcitonin; PLT, Blood platelets; CRP, C-reactive protein; ALT, Alanine transaminase; AST, Aspartate transaminase; HsTnI, High-sensitivity troponin I; BNP, natriuretic protein; IL6, Inter leukin 6.

Survival curves were used to describe the survival status and survival time of the four groups of patients. To compare the survival curves, multiple Cox regression models were used. Adjusted variables were limited to factors such as age, sex, COVID-19 severity, basic illness (hypertension, diabetes, CHD, COPD, CVD, or tumors), high-sensitivity CRP, the lymphocyte count, D-dimer, high sensitivity troponin I (HsTnI) and LDH. These covariates have been commonly adopted in previous studies [1, 4, 18].

All statistical analyses were performed using R software (version 4.1.1; R Foundation, Vienna, Austria), with statistical significance set as a 2-sided *P*<0.05.

## RESULTS

### Baseline characteristics

A total of 1077 patients admitted to the west campus of Wuhan Union Hospital with COVID-19 from January 16 to April 16, 2020, were included in our study. The mean age of the patients was 58.0 years, and the number of male patients was 562 (52.2%). The average length of hospitalization (including the death cases) was 22.3 days. Severe COVID-19 patients numbered 352 (32.7%), and critically severe patients numbered 139 (12.9%). A total of 317 patients (29.4%) had underlying hypertension, and 200 (18.6%) had diabetes. Other comorbidities included coronary heart disease (CHD) (105; 9.7%), tumors (41; 3.8%), chronic obstructive pulmonary disease (COPD) (23; 2.1%), and cerebrovascular disease (CVD) (22; 2.0%).

### AKI in COVID-19 patients

Baseline characteristics: Sixty (5.6%) COVID-19 patients were diagnosed with AKI during hospitalization, 46 (76.7%) of whom were male and 40 (66.7%) of whom were critically severe COVID-19 patients. The mean age of COVID-19 patients with AKI was 67.0 years, which was markedly older than that of patients without AKI (57.5 years). The proportion of critically severe patients was also significantly higher among COVID-19 patients with AKI (40/60; 66.7%) than in patients without AKI (99/1017; 9.7%). Higher proportions of diabetes (18/60; 30%), COPD (5/60; 8.3%) and CVD (4/60; 6.7%) were also observed in COVID-19 patients with AKI than in those without AKI (17.9% (182/1017), 1.8% (18/1017) and 1.8% (18/1017), respectively). The prevalence of other complications was also significantly higher in COVID-19 patients with AKI than in those without AKI, such as respiratory failure, acute respiratory distress syndrome (ARDS), alimentary tract hemorrhage, acute liver dysfunction, acute myocardial injury, heart failure, cerebrovascular accident and disseminated intravascular coagulation (DIC) (Table 1).

Laboratory examination revealed the following: Inflammatory indicators: Compared with patients without AKI, COVID-19 patients with AKI had significantly higher white blood cell (WBC) (8.4*10^9^/L vs. 6.5*10^9^/L) and neutrophil (7.0*10^9^/L vs. 5.0*10^9^/L) counts but lower lymphocyte (0.9*10^9^/L vs. 1.8*10^9^/L) and platelet (PLT) (204.5*10^9^/L vs. 229.0*10^9^/L) counts. The mean values of C-reactive protein (CRP), interleukin-6 (IL-6) and procalcitonin (PCT) were 71.3 mg/L, 385.4 pg/mL and 3.2 ng/mL, respectively, in COVID-19 patients with AKI. These values were significantly higher than those in patients without AKI (26.3 mg/L, 21.8 pg/mL, and 0.2 ng/mL, respectively).

**Table 3:** Treatments during hospitalization of 1077 patients between AKI and non-AKI group.

Variables	Non-AKI (n=1017)	AKI (n=60)	Total (n=1077)	*P*-value	Missing
Antiviral	953/1015 (93.9%)	58/60 (96.7%)	1011/1075 (94.0%)	0.547	2
Antibiotic	714/1015 (70.3%)	54/60 (90.0%)	768/1075 (71.4%)	0.001	2
Glucocorticoids	256/1014 (25.2%)	32/59 (54.2%)	288/1073 (26.8%)	<0.001	4
Gamma globulin	148/1014 (14.6%)	28/59 (47.5%)	176/1073 (16.4%)	<0.001	4
Traditional Chinese medicine	903/1015 (89.0%)	41/59 (69.5%)	944/1074 (87.9%)	<0.001	3
RRT	9/1015 (0.9%)	11/60 (18.3%)	20/1075 (1.9%)	<0.001	2
High flow nasal catheter oxygen inhalation	239/1014 (23.6%)	27/60 (45%)	266/1074 (24.8%)	<0.001	3
Non-invasive ventilation	83/1013 (8.2%)	26/60 (43.3%)	109/1073 (10.2%)	<0.001	4
Invasive mechanical ventilation	42/1014 (4.1%)	26/60 (43.3%)	68/1074 (6.3%)	<0.001	3
ECMO	3/1014 (0.3%)	3/60 (5.0%)	6/1074 (0.6%)	0.003	3
Convalescent plasma	7/1013 (0.7%)	0/60 (0%)	7/1073 (0.7%)	>0.999	4

RRT, renal replacement therapy; EMCO, Extracorporeal membrane oxidation.

**Table 4:** Binary Logistic regression results of AKI.

Variables	Level	Pool estimates based on imputed datasets (n=1077)				Estimates based on complete dataset (n=820)			
		OR	95%CI		*p*	OR	95%CI		*p*
			Lower	Upper			Lower	Upper	
Respiratory failure	No	Ref				Ref			
	Yes	7.08	3.36	14.93	<0.001	3.91	1.53	10.00	<0.001
Acute myocardial injury	No	Ref				Ref			
	Yes	4.18	1.91	9.15	<0.001	3.54	1.31	9.59	0.013
Peak creatinine (μmol/L)		1.01	1.01	1.02	<0.001	1.03	1.02	1.03	<0.001
PCT (ng/mL)		1.20	1.04	1.40	0.014	1.21	1.03	1.41	0.017

PCT: Procalcitonin.

Biochemical indicators: The albumin concentration was significantly reduced in COVID-19 patients with AKI (29.6 g/L) compared with those without AKI (33.9 g/L). COVID-19 patients with AKI had higher blood potassium levels than those without AKI (4.2 mmol/L vs. 4.1 mmol/L). Hypocalcemia was more obvious in COVID-19 patients with AKI, and the mean serum calcium was 1.9 mmol/L compared with 2.1 mmol/L in patients without AKI. Among COVID-19 patients with AKI, 66.7% (18/27) had hematuria, 53.8% (14/26) had proteinuria, 32.8% (77/235) of patients without AKI had hematuria, and 11.6% (27/232) had proteinuria. The average baseline creatinine level was 126.0 µmol/L in COVID-19 patients with AKI, a value that was significantly higher than that of their non-AKI counterparts, 71.2 µmol/L. A similar pattern was observed in the average peak creatinine level between COVID-19 patients with AKI (219.6 µmol/L) and those without AKI (74.3 µmol/L). Serum urea nitrogen levels were also markedly increased in patients with AKI, with an average of 12.3 mmol/L. These values were closely associated with renal function in COVID-19 patients with AKI, indicating that AKI patients had kidney damage on admission (Tables 1 and 2).

Coagulation function: The prothrombin time (PT) (15.2 s vs. 13.9 s) and activated partial thromboplastin time (APTT) (39.7 s vs. 38.1 s) were significantly longer in COVID-19 patients with AKI than in those without AKI, and D-dimer (3.7 µg/mL vs. 1.4 µg/mL) levels were significantly higher in patients with AKI (Table 2).

**Figure 1.**
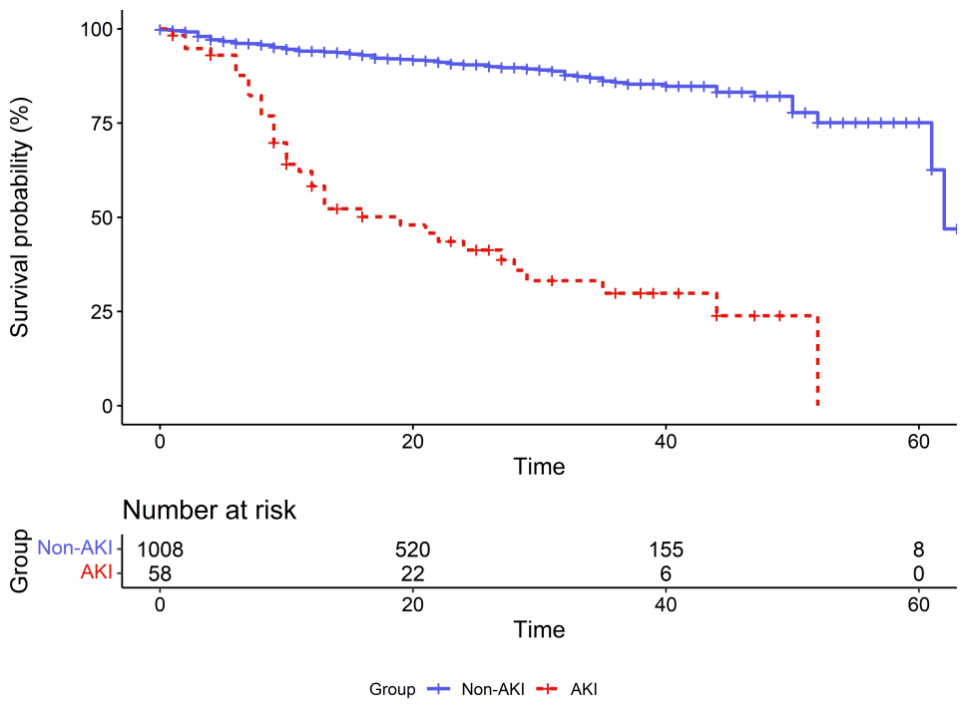
Survival curves for AKI and non-AKI patients.

**Table 5:** The results of multivariate cox regression model among patients with different AKI and CKD status.

Variables	Level	Pool estimates based on imputed datasets (n=1066)				Estimates based on complete dataset (n=507)			
		HR	95%CI		*p*	HR	95%CI		*p*
			Lower	Upper			Lower	Upper	
Non-AKI/CKD		Ref				Ref			
AKI only		3.39	2.06	5.57	<0.001	4.04	2.17	7.53	<0.001
CKD only		1.28	0.71	2.30	0.411	1.50	0.63	3.56	0.355
AKI/CKD		4.20	2.19	8.06	<0.001	6.15	2.76	13.73	<0.001
Sex	Female	Ref				Ref			
	Male	1.10	0.76	1.60	0.619	1.21	0.68	2.16	0.518
Age		1.00	0.99	1.02	0.552	1.00	0.98	1.02	0.802
COVID-19 severity	Mild or Moderate	Ref				Ref			
	Severe or critically severe	2.43	1.46	4.03	<0.001	3.55	1.36	9.29	0.010
Basic illness	No	Ref				Ref			
	Yes	0.94	0.65	1.34	0.718	1.22	0.72	2.06	0.461
Lymphocyte count		0.74	0.50	1.09	0.129	0.62	0.34	1.16	0.135
CRP		1.01	1.00	1.01	<0.001	1.01	1.01	1.02	<0.001
LDH		1.00	1.00	1.00	0.016	1.00	1.00	1.00	0.178
D-dimer		1.06	0.99	1.13	0.092	1.08	0.99	1.17	0.088
HsTnI		1.00	1.00	1.00	0.769	1.00	1.00	1.00	0.174

Note: Basic illness yes means the patient has one or more of the following basic illnesses: Hypertension, Diabetes, CHD, COPD, CVD, Tumor.

The treatments were as follows: Assisted ventilation: After admission to the hospital, the need for oxygen inhalation through nasal catheters in COVID-19 patients with AKI was substantially increased and reached 45% (27/60), which was significantly higher than 23.6% (239/1014) of the patients without AKI. COVID-19 patients with AKI who required invasive and noninvasive mechanical ventilation accounted for 43.3% (26/60) in both groups, a proportion that was significantly higher than the ventilation needs of the patients without AKI (8.2% (83/1013) and 4.1% (42/1014), respectively).

Medications: The proportions of COVID-19 patients hospitalized with AKI using glucocorticoids and gamma-globulin were 54.2% and 47.5%, respectively, compared with 25.2% and 14.6%, respectively, in patients without AKI (p<0.05).

Blood purification: The use of extracorporeal membrane oxygenation (ECMO) reached 5% (3/60) in COVID-19 patients with AKI, a proportion that was significantly higher than 0.3% (3/1014) in patients without AKI. The proportions of renal replacement therapy (RRT) were 18.3% (11/60) and 0.9% (9/1015) in patients with AKI and those without AKI, respectively (Table 3).

The prognosis was as follows: A total of 28.3% of COVID-19 patients with AKI were admitted to the intensive care unit (ICU), a proportion that was significantly higher than 3.4% of patients without AKI. The death rate of patients without AKI was 10.7% (109/1017); compared with these patients, COVID-19 patients with AKI had a worse prognosis, with a fatality rate of 65% (39/60) (Table 1). The rate of renal recovery at discharge for COVID-19 patients with AKI was 73.3% (Table 7). Figure 1 shows the survival curves between COVID-19 patients with different AKI statuses. In the multivariate Cox model with all the covariates meeting the proportional hazard assumption, the adjusted hazard ratio (HR) for AKI was 3.44 (95% CI, 2.26-5.24), suggesting a higher fatality and shorter survival time for COVID-19 patients with AKI than for those without AKI (Table 6).

### Association between the severity of AKI and COVID-19

Among the 60 COVID-19 patients with AKI, patients with stage 1, 2, or 3 AKI numbered 38, 7, or 15, respectively. The proportions of critically severe COVID-19 patients with stage 1-3 AKI were 55.3% (21/38), 71.4% (5/7) and 93.3% (14/15), respectively. We also observed a higher ICU rate and worse prognosis in patients with more severe AKI. For example, the ICU rates of COVID-19 patients with stage 1, 2, and 3 AKI were 15.8% (6/38), 42.9% (3/7) and 53.3% (8/15), respectively, whereas the fatality rates for the three groups were 55.3% (21/38), 57.1% (4/7) and 93.3% (14/15), respectively (Table 7).

**Table 6:** The results of multivariate cox regression model between AKI and Non-AKI groups.

Variables	Level	Pool estimates based on imputed datasets (n=1066)				Estimates based on complete dataset (n=507)			
		HR	95%CI		*p*	HR	95%CI		*p*
			Lower	Upper			Lower	Upper	
AKI	No	Ref				Ref			
	Yes	3.44	2.26	5.24	<0.001	4.15	2.45	7.01	<0.001
Sex	Female	Ref				Ref			
	Male	1.12	0.77	1.62	0.559	1.28	0.73	2.27	0389
Age		1.00	0.99	1.02	0.556	1.00	0.98	1.02	0.866
COVID-19 severity	Mild or Moderate	Ref				Ref			
	Severe or critically severe	2.41	1.45	4.00	0.001	3.44	1.32	8.97	0.012
Basic illness	No	Ref				Ref			
	Yes	0.96	0.68	1.37	0.833	1.28	0.76	2.14	0.357
lymphocyte count		0.74	0.50	1.10	0.139	0.66	0.36	1.21	0.180
CRP		1.01	1.00	1.01	<0.001	1.01	1.01	1.02	<0.001
LDH		1.00	1.00	1.00	0.003	1.00	1.00	1.00	0.049
D-dimer		1.06	0.99	1.13	0.085	1.08	1.00	1.18	0.063
HsTnI		1.00	1.00	1.00	0.873	1.00	1.00	1.00	0.265

Note: Basic illness yes means the patient has one or more of the following basic illnesses: Hypertension, Diabetes, CHD, COPD, CVD, Tumor.

**Figure 2.**
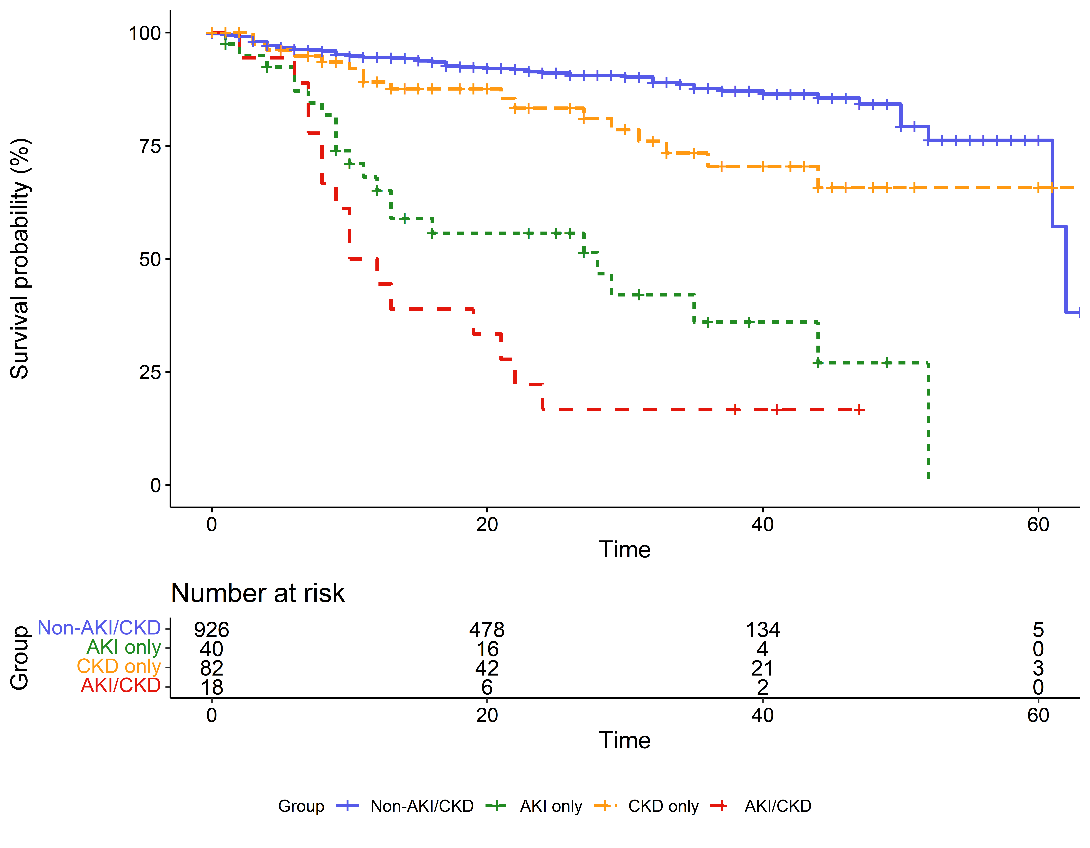
Survival curves of patients with different AKI and CKD statuses.

**Table 7:** General characteristics and renal replacement of 60 patients with different AKI levels.

Variables	AKI (n=60)				*P-value*	*Missing*
	AKI-1 (n=38)	AKI-2 (n=7)	AKI-3 (n=15)	Total (n=60)		
COVID-19 Severity						
moderate	10/38 (26.3%)	0/7 (0%)	0/15 (0%)	10/60 (16.7%)	0.037	0
severe	7/38 (18.4%)	2/7 (28.6%)	1/15 (6.7%)	10/60 (16.7%)		
Critically severe	21/38 (55.3%)	5/7 (71.4%)	14/15 (93.3%)	40/60 (66.7%)		
Renal recovery	8/12 (66.7%)	3/3(100%)	0/0 (-)	11/15 (73.3%)	0.516	45
Transferred to ICU	6/38 (15.8%)	3/7 (42.9%)	8/15 (53.3%)	17/60 (28.3%)	0.014	0
Death	21/38 (55.3%)	4/7 (57.1%)	14/15 (93.3%)	39/60 (65%)	0.021	0
Proteinuria	7/15 (46.7%)	0/3 (0%)	7/8 (87.5%)	14/26 (53.8%)	0.017	34
Hematuria	11/16 (68.8%)	1/3 (33.3%)	6/8 (75.0%)	18/27 (66.7%)	0.523	33
RRT	3/38 (7.9%)	2/7 (28.6%)	6/15 (40.0%)	11/60 (18.3%)	0.013	0
RRT mode						
CVVH	0/2 (0%)	0/2 (0%)	1/5 (20.0%)	1/9 (11.1%)	>0.999	51
CRRT	2/2 (100%)	2/2 (100%)	4/5 (80.0%)	8/9 (88.9%)		
RRT frequency						
≤1 times/week	0/2 (0%)	0/1 (0%)	1/5 (20.0%)	1/8 (12.5%)	0.238	52
1-2 times/week	0/2 (0%)	0/1 (0%)	2/5 (40.0%)	2/8 (25.0%)		
2-3 times/week	0/2 (0%)	1/1 (100%)	0/5 (0%)	1/8 (12.5%)		
≥4 times/week	2/2 (100%)	0/1 (0%)	2/5 (40.0%)	4/8 (50.0%)		
Renal replacement time						
2<-4 h	1/2 (50.0%)	0/1 (0%)	0/3 (0%)	1/6 (16.7%)	>0.999	54
4<-6 h	1/2 (50.0%)	1/1 (100%)	2/3 (66.7%)	4/6 (66.7%)		
6<-8 h	0/2 (0%)	0/1 (0%)	1/3 (33.3%)	1/6 (16.7%)		

RRT, renal replacement therapy; CVVH, Continuous Veno Venous Hemofiltration; CRRT, Continuous renal replacement therapy.

COVID-19 patients with more severe AKI required more RRT, and the percentages of patients receiving RRT were 7.9% (3/38), 28.6% (2/7), and 40% (6/15) in AKI stage 1, 2 and 3 patients, respectively. Specific information on RRT in AKI patients with COVID-19 is presented in Table 7.

### Different AKI and CKD statuses in COVID-19 patients

COVID-19 patients were further divided into four groups based on the presence of AKI or CKD: AKI on CKD (AKI/CKD, AKI occurred based on CKD) (n=18); AKI only (AKI patients without CKD) (n=42); CKD only (CKD patients without AKI) (n=83); and non-AKI/CKD (patients without AKI or CKD) (n=934). Of the 101 (10.2%) patients with CKD, 18 (17.8%) developed AKI. The percentage of patients with diabetes in the AKI/CKD group was 50% (9/18), which was significantly higher than that of patients with AKI only (9/42, 21.4%), CKD only (31/83, 37.3%), or neither (151/934, 16.2%). Additionally, the prevalence of complications, such as ARDS, alimentary tract hemorrhage, heart failure and DIC, was significantly higher in the AKI/CKD group than in the remaining three groups (Table 8).

Laboratory tests also showed that COVID-19 patients with AKI/CKD had higher levels of uric acid and blood glucose and a higher incidence of proteinuria and hematuria than the other three groups. The mean peak creatinine level during hospitalization was significantly higher in the AKI/CKD group (271.7 µmol/L) than in the other three groups (AKI only, 197.3 µmol/L; CKD only, 121.6 µmol/L; non-AKI/CKD, 70.2 µmol/L) (Table 9).

The prognosis of AKI/CKD group was significantly worse than that of the other three groups. The fatalities were 83.3%, 57.1%, 22.9%, and 9.6% in the AKI/CKD, AKI only, CKD only, and non-AKI/CKD groups, respectively. The survival curves for the four groups are shown in Figure 2. In the multivariate Cox model using all the covariates that met the proportional hazard assumption, the adjusted HRs for patients with AKI/CKD and those with AKI only were 4.20 (95% CI, 2.19-8.06) and 3.39 (95% CI, 2.06-5.57), respectively, referring to the non-AKI/CKD group. The results indicated a higher fatality and shorter survival time for COVID-19 patients in the AKI/CKD group and AKI only group (Table 5).

**Table 8:** General characteristics of 1077 patients with different AKI and CKD status.

Variables	Non-CKD (n=976)		CKD (n=101)		Total (n=1077)	*P*-value	*Missing*
	Non-AKI (n=934)	AKI (n=42)	Non-AKI (n=83)	AKI (n=18)			
age	56.9±14.1	67.1±13.3	63.8±12.1	66.6±16.8	58.0±14.3	<0.001	0
Gender						<0.001	0
Female	469/934 (50.2%)	10/42 (23.8%)	32/83 (38.6%)	4/18 (22.2%)	515/1077 (47.8%)		
Male	465/934 (49.8%)	32/42 (76.2%)	51/83 (61.4%)	14/18 (77.8%)	562/1077 (52.2%)		
Hospitalization time (day)	22.4±13.8	18.9±14.2	24.8±16.7	16.9±13.1	22.3±14.1	0.050	11
Systolic BP (mm Hg)	130.9±17.5	132.9±21.6	134.7±21.7	133.7±23.9	131.4±18.2	0.297	69
Diastolic BP (mm Hg)	81.8±12.5	78.9±11.0	80.7±16.3	79.2±16.9	81.5±12.8	0.549	72
Basic illness							
Hypertension	243/934 (26%)	10/42 (23.8%)	53/83 (63.9%)	11/18 (61.1%)	317/1077 (29.4%)	<0.001	0
Diabetes	151/934 (16.2%)	9/42 (21.4%)	31/83 (37.3%)	9/18 (50%)	200/1077 (18.6%)	<0.001	0
CHD	84/934 (9%)	7/42 (16.7%)	13/83 (15.7%)	1/18 (5.6%)	105/1077 (9.7%)	0.085	0
COPD	16/934 (1.7%)	5/42 (11.9%)	2/83 (2.4%)	0/18 (0%)	23/1077 (2.1%)	0.005	0
Cerebrovascular disease	14/934 (1.5%)	2/42 (4.8%)	4/83 (4.8%)	2/18 (11.1%)	22/1077 (2%)	0.005	0
Tumor	34/934 (3.6%)	3/42 (7.1%)	3/83 (3.6%)	1/18 (5.6%)	41/1077 (3.8%)	0.418	0
Transferred to ICU	24/934 (2.6%)	12/42 (28.6%)	11/83 (13.3%)	5/18 (27.8%)	52/1077 (4.8%)	<0.001	0
Death	90/934 (9.6%)	24/42 (57.1%)	19/83 (22.9%)	15/18 (83.3%)	148/1077 (13.7%)	<0.001	0
Complications							
Respiratory failure	81/934 (8.7%)	25/42 (59.5%)	20/83 (24.1%)	17/18 (94.4%)	143/1077 (13.3%)	<0.001	0
Gastrointestinal bleeding	10/934 (1.1%)	3/42 (7.1%)	4/83 (4.8%)	5/18 (27.8%)	22/1077 (2%)	<0.001	0
Acute liver dysfunction	284/933 (30.4%)	23/42 (54.8%)	34/83 (41%)	15/18 (83.3%)	356/1076 (33.1%)	<0.001	1
Acute myocardial injury	38/931 (4.1%)	21/42 (50%)	11/83 (13.3%)	11/17 (64.7%)	81/1073 (7.5%)	<0.001	4
Heart failure	28/934 (3%)	10/42 (23.8%)	6/83 (7.2%)	13/18 (72.2%)	57/1077 (5.3%)	<0.001	0
ARDS	42/933 (4.5%)	14/42 (33.3%)	12/83 (14.5%)	16/18 (88.9%)	84/1076 (7.8%)	<0.001	1
Cerebrovascular accident	8/933 (0.9%)	3/42 (7.1%)	1/83 (1.2%)	1/18 (5.6%)	13/1076 (1.2%)	0.008	1
Bacteremia	10/934 (1.1%)	3/42 (7.1%)	5/83 (6%)	3/18 (16.7%)	21/1077 (1.9%)	<0.001	0
DIC	6/933 (0.6%)	4/42 (9.5%)	3/83 (3.6%)	7/18 (38.9%)	20/1076 (1.9%)	<0.001	1
hematuria	39/178 (21.9%)	8/15 (53.3%)	38/57 (66.7%)	10/12 (83.3%)	95/262 (36.3%)	<0.001	815
proteinuria	5/177 (2.8%)	5/15 (33.3%)	22/55 (40%)	9/11 (81.8%)	41/258 (15.9%)	<0.001	819
ACEI/ARB history	37/677 (5.5%)	0/33 (0%)	8/65 (12.3%)	2/13 (15.4%)	47/788 (6%)	0.024	289

BP, blood pressure; Admission to outcome(day): Refers to the time from hospital admission to cure discharge or died. CKD, Chronic kidney disease; CHD, coronary heart disease; COPD, Chronic obstructive pulmonary disease; AKI, acute kidney injury; CVD, cerebrovascular disease; ARDS, Acute Respiratory Distress Syndrome; DIC, disseminated intravascular coagulation; LDH, Lactate dehydrogenase; UA, uric acid; PCT, Procalcitonin; SO2, Blood oxygen saturation; PLT, Blood platelets; CRP, C-reactive protein.

**Table 9:** Laboratory examination of 1077 patients among patients with different AKI and CKD status.

Variables	Non-CKD (n=976)		CKD (n=101)		Total (n=1077)	*P*-value	Missing
	Non-AKI (n=934)	AKI (n=42)	Non-AKI (n=83)	AKI (n=18)			
Hemoglobin (g/L)	126.4±19.1	128.3±22.1	126.9±21.0	127.8±18.2	126.5±19.4	0.784	8
PLT (10^9^/L)	230.7±85.7	201.6±88.9	210.5±79.0	211.1±98.9	227.6±85.9	0.012	7
WBC count (10^9^/L)	6.4±3.0	7.8±4.1	7.7±5.5	9.6±5.1	6.6±3.4	<0.001	9
Neutrophil count (10^9^/L)	5.0±14.3	6.4±4.1	6.0±4.8	8.5±5.01	5.1±13.4	<0.001	11
Lymphocyte count (10^9^/L)	1.6±4.7	0.9±0.5	4.8±23.1	0.74±0.63	1.8±7.8	<0.001	8
Eosinophil count(10^9^/L)	0.2±1.2	0±0.1	0.1±0.1	0±0.1	0.1±1.1	<0.001	7
Albumin (g/L)	34.1±6.3	29.4±5.1	32.0±6.1	30.1±5.2	33.7±6.3	<0.001	6
Globulin (g/L)	30.5±9.6	33.7±7.7	36.6±36.8	34.9±6.4	31.2±13.8	<0.001	13
ALT (U/L)	44.1±41.6	56.2±43.6	39.6±46.0	51.9±53.0	44.4±42.2	0.049	5
AST (U/L)	33.3±24.1	69.8±87.5	37.2±37.7	49.3±40.3	35.3±31.4	<0.001	5
LDH (U/L)	245.5±125.1	436.0±197.6	355.2±283.9	462.9±178.9	265.1±156.5	<0.001	11
CK (U/L)	95.4±128.8	347.8±398.2	248.1±482.6	216.0±426.8	119.3±212.7	<0.001	126
CK-MB (U/L)	12.1±8.5	19.2±15.5	17.4±15.9	18.7±9.1	12.9±9.8	<0.001	111
HsTnI (ng/L)	49.3±516.8	156.5±298.5	96.3±469.5	420.4±1169.3	69.4±535.5	<0.001	479
BNP (pg/mL)	71.1±222.3	157.5±290.9	81.0±129.3	191.6±152.7	81.4±218.7	<0.001	593
Admission creatinine (μmol/L)	66.9±21.6	124.0±136.2	119.0±194.0	130.6±89.7	74.2±66.7	<0.001	6
Peak creatinine (μmol/L)	70.2±22.2	197.3±150.3	121.6±196.1	271.7±143.1	82.6±77.0	<0.001	26
Serum urea nitrogen (mmol/L)	4.9±2.2	12.1±13.4	9.1±7.4	12.8±7.4	5.7±4.4	<0.001	7
UA(μmol/L)	272.0±103.6	333.1±252.1	332.7±144.3	394.9±154.7	281.2±119.7	<0.001	12
Blood natrium (mmol/L)	141.7±59.8	141.8±8.1	139.2±4.4	136.5±6.9	141.4±55.7	0.033	13
Potassium (mmol/L)	4.1±4.8	4.1±0.7	4.0±0.5	4.4±0.7	4.1±4.4	0.021	12
Blood calcium (mmol/L)	2.2±4.3	1.9±0.1	2.0±0.2	2.0±0.2	2.1±4.0	<0.001	10
Fasting blood glucose (mmol/L)	7.3±23.5	7.9±3.3	8.5±4.1	13.1±8.2	7.5±22.0	<0.001	19
PT (s)	13.8±7.2	15.7±8.6	15.7±12.7	14.0±1.3	14.0±7.8	0.003	65
APTT (s)	38.0±21.3	41.1±8.6	38.4±8.6	36.5±4.6	38.2±20.0	0.004	66
D-dimer (ug/mL)	1.4±2.2	3.2±3.2	2.1±2.9	4.9±3.2	1.6±2.4	<0.001	149
High-sensitivity CRP (mg/L)	25.3±37.2	66.5±51.5	38.8±47.3	84.6±51.0	29.0±40.6	<0.001	124
PCT (ng/mL)	0.2±0.8	1.4±3.9	0.4±1.7	7.8±25.5	0.4±3.7	<0.001	240
IL6 (pg/mL)	22.7±262.5	499.3±1494.3	11.1±21.7	72.3±115.9	35.1±348.9	<0.001	666

LDH, Lactate dehydrogenase; UA, Uric acid; PCT: Procalcitonin; PLT, Blood platelets; CRP, C-reactive protein; ALT, Alanine transaminase; AST, Aspartate transaminase; HsTnI, High-sensitivity troponin; BNP, natriuretic protein; IL6, Inter leukin 6.

### Risk factors for AKI in COVID-19 patients

Multiple logistic regression was performed to detect risk factors for AKI in COVID-19 patients. The results indicated that the presence of complications, such as respiratory failure, acute myocardial injury, a higher hospitalization creatinine level and an increased PCT level, were independent risk factors for the development of AKI in COVID-19 patients (Table 4).

## DISCUSSION

In the current study, the incidence of AKI in COVID-19 patients was 5.6%, which is similar to earlier reports (3-6.6%) from other hospitals in Wuhan [1, 4, 7, 23]. The development of AKI in COVID-19 disease may be related to the expression of ACE2 receptors in the kidney used by SARS-CoV-2 to enter host cells [24-26]. The ACE2 protein is highly expressed in tubular epithelial cells that are damaged by the virus [27, 28]. Factors other than SARS-CoV-2 virulence contribute to AKI in COVID-19 patients, such as systemic hypoxia, prothrombotic state, and possible drug or hyperventilation-relevant rhabdomyolysis [14, 29]. In our study, the AKI incidence rates were 1.8%, 2.8% and 28.8% in mild, severe and critically severe COVID-19 patients, respectively. The proportion of severe COVID-19 patients with AKI was significantly higher than that of patients without AKI, and the proportion of severe COVID-19 patients increased with AKI severity, indicating that severe patients were more likely to develop AKI, a finding that is consistent with the conclusions of previous studies [1, 4]. Among the COVID-19 patients, AKI was more likely to occur in male patients, the elderly, patients with more severe disease states and those with comorbidities, such as hypertension, diabetes, CHD, COPD, and CKD, findings that are also consistent with those of a previous study [1, 4, 13]. Additionally, COVID-19 patients with AKI were more likely to develop respiratory failure, gastrointestinal bleeding, acute liver injury, acute myocardial injury, heart failure, and ARDS, reflecting a more severe condition of COVID-19 patients with AKI. The fatality of all COVID-19 patients in our study was 13.7%, which is much higher than the reported fatalities among all COVID-19 cases in Wuhan (5.0%) and elsewhere in China (2.4%) [30, 31]. This finding may have contributed to the increased proportion of severe patients (32.7%) and critically severe patients (12.9%) admitted to the study hospital. The fatality rates of COVID-19 patients with AKI reported in different studies vary widely, from 35% to 88.9% [1, 13]. In our study, the fatality of COVID-19 patients with AKI reached 65%, which was much higher than that of patients without AKI (10.7%). The fatality of COVID-19 patients also increased with AKI severity. COVID-19 patients with AKI had a worse prognosis, likely because of the abrupt loss of kidney function and more severe COVID-19 infection. However, the rate of renal recovery at the discharge for COVID-19 patients with AKI was 73.3%, indicating that renal function can be restored when COVID-19 infection is controlled in most patients.

**Table 10:** Treatments during hospitalization of 1077 patients among patients with different AKI and CKD status.

Variables	Non-CKD (n=976)		CKD (n=101)		Total (n=1077)	*P*-value	Missing
	Non-AKI (n=934)	AKI (n=42)	Non-AKI (n=83)	AKI (n=18)			
Antiviral	872/932 (93.6%)	40/42 (95.2%)	81/83 (97.6%)	18/18 (100%)	1011/1075 (94%)	0.444	2
Antibiotic	644/932 (69.1%)	36/42 (85.7%)	70/83 (84.3%)	18/18 (100%)	768/1075(71.4%)	<0.001	2
Glucocorticoids	225/931 (24.2%)	21/41 (51.2%)	31/83 (37.3%)	11/18 (61.1%)	288/1073(26.8%)	<0.001	4
Gamma globulin	128/931 (13.7%)	18/41 (43.9%)	20/83 (24.1%)	10/18 (55.6%)	176/1073(16.4%)	<0.001	4
Traditional Chinese medicine	829/932 (88.9%)	31/41 (75.6%)	74/83 (89.2%)	10/18 (55.6%)	944/1074(87.9%)	<0.001	3
RRT	6/932 (0.6%)	7/42 (16.7%)	3/83 (3.6%)	4/18 (22.2%)	20/1075 (1.9%)	<0.001	2
High flow nasal catheter oxygen inhalation	217/931 (23.3%)	18/42 (42.9%)	22/83 (26.5%)	9/18 (50%)	266/1074(24.8%)	0.002	3
Non-invasive ventilation	67/930 (7.2%)	16/42 (38.1%)	16/83 (19.3%)	10/18 (55.6%)	109/1073(10.2%)	<0.001	4
Invasive mechanical ventilation	31/931 (3.3%)	15/42 (35.7%)	11/83 (13.3%)	11/18 (61.1%)	68/1074 (6.3%)	<0.001	3
ECMO	3/931 (0.3%)	3/42 (7.1%)	0/83 (0%)	0/18 (0%)	6/1074 (0.6%)	0.004	3
Convalescent plasma	7/930 (0.8%)	0/42 (0%)	0/83 (0%)	0/18 (0%)	7/1073 (0.7%)	>0.999	4

RRT, Renal replacement therapy; EMCO, Extracorporeal membrane oxidation.

Worse laboratory indicators were observed in COVID-19 patients with AKI. Compared with patients without AKI, COVID-19 patients with AKI in our study had lower platelet counts, lymphocyte counts, albumin levels and serum calcium levels but had higher leukocyte counts, neutrophil counts and serum potassium levels. A longer PT, a longer APTT and a higher D-dimer level were also observed in COVID-19 patients with AKI than in those without AKI. These findings indicate that impaired coagulation, immune function and kidney function in COVID-19 patients with AKI were worse. A previous meta-analysis of 45 studies also found that higher levels of white blood cells, neutrophils and D-dimer and lower lymphocyte and platelet counts were associated with severe COVID-19 disease [32]. Notably, the average creatinine level of AKI patients with COVID-19 admission was 125.97 µmol/L, which was higher than the normal range of creatinine, suggesting that COVID-19 patients with AKI had kidney damage on admission. COVID-19 patients with CKD and AKI onset before admission may also explain this finding.

A total of 101 (10.2%) patients had CKD among all COVID-19 patients, and 18 patients (23.3%) also had AKI. The fatality of COVID-19 patients with AKI/CKD was significantly greater than that of patients with AKI or CKD only. The peak creatinine level during hospitalization was significantly higher in patients with AKI/CKD than in those with AKI or CKD only, indicating that AKI/CKD was more serious. AKI and CKD are interconnected syndromes. AKI contributes to the initiation and progression of CKD. Additionally, CKD predisposes patients to AKI. Therefore, AKI/CKD has a poor prognosis. The underlying mechanism of AKI/CKD is largely unknown. Major pathophysiological changes in CKD may increase the sensitivity or susceptibility to AKI and suppress kidney repair or recovery from AKI in CKD patients [33]. Whether discharged COVID-19 patients with AKI will develop CKD and the rate of this development require further exploration.

Our analysis revealed that independent risk factors for the development of AKI in COVID-19 patients included complications, such as respiratory failure and acute cardiac injury, increased creatinine and elevated PCT. Respiratory failure indicates more severe COVID-19, causing damage to other organs, such as the kidneys. Early detection and treatment of COVID-19 patients with AKI are imperative to identify acute myocardial injury using cardiac enzymes. Creatinine and PCT assessments during hospitalization can also be used to detect AKI. PCT is an inflammatory indicator, and serum PCT levels increase rapidly in inflammatory or infectious states. AKI is closely related to PCT, which has even been disclosed as a predictor of AKI in different clinical settings, irrespective of the existence of infection [34, 35]. PCT was a risk factor for AKI development in COVID-19 patients in the present study, suggesting that clinicians should remain alert for the occurrence of AKI in COVID-19 patients with high PCT levels. CRP and IL-6 levels were also increased in COVID-19 patients with AKI, suggesting that the inflammatory storm was stronger in COVID-19 patients with AKI than in those without AKI. Higher serum IL-6 levels were also a risk factor for AKI in COVID-19 patients in another study [36]. Other predictors of AKI in COVID-19 patients from different studies included the pneumonia severity, increased age, male sex, diabetes mellitus, hypertension, a history of cardiovascular disease, an increased body mass index (BMI), mechanical ventilation, vasopressor medications and a history of treatment with renal-angiotensin-aldosterone inhibiting (RAASi) medications [1, 13, 36].

The present study has several limitations. First, some of the enrolled patients were missing data. Second, although a multiple logistic regression model was conducted with many covariates included, some unmeasured or unknown variables related to AKI in COVID-19 might have been omitted. Third, we only studied the incidence of AKI and AKI/CKD in COVID-19 patients and could not assess the long-term kidney outcomes of COVID-19 patients after hospital discharge. Further research is needed to explore the kidney outcomes of discharged COVID-19 patients and whether they develop CKD and its incidence. Finally, these data were all derived from electronic medical records, and the serum creatinine levels at admission were used to identify AKI. Some patients with AKI at admission may have been omitted because of their comparatively lower creatinine level, leading to an underestimated incidence of AKI in COVID-19 patients.

In conclusion, we found that COVID-19 patients complicated with AKI had overall higher fatality rates and shorter survival times than their non-AKI counterparts. Patients with AKI/CKD and COVID-19 infection had a worse prognosis than COVID-19 patients with AKI only. We also identified that AKI in COVID-19 patients was closely related to respiratory failure, acute myocardial injur creatinine and PCT level. Further study is needed to investigate the long-term outcomes of COVID-19 patients with AKI after discharge from the hospital.
